# Survival Machine-Learning Approach for Predicting Under-Five Mortality in Low Sociodemographic Index States of India

**DOI:** 10.34172/jrhs.9033

**Published:** 2025-06-10

**Authors:** Mukesh Vishwakarma, Gargi Tyagi, Rehana Vanaja Radhakrishnan

**Affiliations:** ^1^Department of Mathematics and Statistics, Faculty of Mathematics and Computing, Banasthali Vidyapith, Rajasthan, India; ^2^Administrative Office, Sarojini Naidu Medical College, Agra, Uttar Pradesh, India; ^3^Department of Community Medicine, Andaman and Nicobar Islands Institute of Medical Sciences, Sri Vijaya Puram, India

**Keywords:** Child mortalities, Random forest, Survival analyses, Cox models, ROC curves

## Abstract

**Background:** Each year, millions of children under five die globally, with many of these deaths being preventable. The situation is particularly concerning in low sociodemographic index (LSDI) states of India, where the under-five mortality rate is 45 children per 1000 live births. This study aimed to predict under-five mortality and determine related key factors.

**Study Design:** A cross-sectional study.

**Methods:** This study analyzed National Family Health Survey-5 (NFHS-5) data related to 94,202 children from the LSDI states of India. Several survival models were tested, including Cox proportional hazards, random survival forest, and gradient-boosted survival, to identify factors linked to child mortality. Model performance was evaluated using metrics such as the concordance index, integrated Brier score, and time-dependent receiver operating characteristic (ROC) curves.

**Results:** Among the studied children, 4.5% (4,284) died before their fifth birthday. The risk of death was higher in children born to younger (15–25 years) mothers (hazard ratio [HR] = 1.113, 95% confidence interval (CI): 1.034, 1.198; *P* < 0.001), uneducated mothers (HR = 1.263, 95% CI: 1.098–1.454; *P* < 0.0001), mothers with a poorer wealth index (HR = 1.719, 95% CI: 1.475–2.003; *P* < 0.0001), and children with low birth weight (HR = 2.091, 95% CI: 1.934–2.26; *P* < 0.001). The random survival forest model outperformed in identifying these risk factors.

**Conclusion:** This study highlights the importance of empowering women through education, improving family planning, addressing poverty, and providing equitable healthcare to reduce child mortality. These insights can help shape policies and initiatives to improve the survival and health of children in vulnerable communities.

## Background

 Under-five mortality is a crucial indicator used to assess the health status of a country and its people.^1^ As defined by the World Health Organization, under-five mortality refers to the total number of deaths of children that occur within the first five years of life per 1000 live births.^2^ It is a key measure of progress for sustainable development goals (SDGs). SDG 3.2 aims to eliminate avoidable deaths of children under the age of five and reduce the under-five mortality rate (U5MR) to 25 per 1000 live births or less in all countries by 2030.^3^ Worldwide, the overall number of deaths among children under the age of 5 has decreased from 12.8 million in 1990 to 4.9 million in 2022. The global U5MR has reduced by 61% since 1990, declining from 94 deaths per 1000 live births in 1990 to 37 in 2023.^4^ Nonetheless, the burden of this tragic death toll falls heavily on families in sub-Saharan Africa and Southern Asia, especially in low-income and lower-middle-income nations. Although only three out of five live births occur in these regions, they account for four out of five deaths among children under five. Southern Asia alone contributes to 26% of the world’s U5MR.^5^ Newborn infections, congenital impairments, premature birth, malaria, pneumonia, sepsis, measles, delivery complications, and diarrhoea are all well-established preventable factors leading to death in children under the age of five.^6,7^ In India, under-five mortality has been a significant health concern, with 109 deaths per 1000 live births in 1992–1993, declining to 41.9 per 1000 live births in 2019–2021, according to the National Family Health Survey (NFHS).^8,9^ However, in low sociodemographic index (LSDI) states (i.e., Uttar Pradesh, Bihar, Rajasthan, Madhya Pradesh, Chhattisgarh, and Jharkhand), the U5MR remains higher than the national average, at approximately 45 deaths per 1000 live births. These states require targeted interventions to identify and address the underlying factors contributing to child mortality.

 Over the past few decades, data analysts have relied on various survival analysis methods, including Cox regression and parametric regression models, to study survival data. While these models are straightforward and offer good interpretability, medical data often involve complex, multidimensional, and non-linear relationships. As a result, traditional statistical techniques may not be sufficient for accurately predicting child mortality. To address this challenge, survival machine-learning (ML) techniques have emerged as a powerful alternative, demonstrating superior performance in handling non-linear and complex datasets.

 Multiple studies have evaluated classification ML algorithms in predicting child mortality and other outcomes of various diseases against other prediction models, and survival ML models have been employed to predict cancer outcomes. To the best of our knowledge, no research has so far been undertaken to predict child mortality utilizing survival ML algorithms. Thus, this study aims to compare various survival analysis techniques, ranging from traditional statistical models to state-of-the-art ML algorithms, in predicting child mortality and determining factors affecting it.

## Methods

 This study utilized unit-level data obtained from the NFHS conducted from 2019 to 2021. It was a cross-sectional, large-scale, multi-round survey performed in a representative sample of households throughout India. It is a comprehensive demographic and health study covering all 707 districts of India. In this study, the LSDI states of India with 248 districts were taken into consideration. It comprised 104 692 samples at the time of the survey, and the analysis was conducted on 94 202 samples after removing missing values from one or more variables under consideration.

###  Sociodemographic index

 It was calculated by taking the geometric mean of education attainment, the total fertility rate of the region or country, and per capita income, developed by Global Burden of Disease (GBD) researchers. It serves as a tool to gauge the level of economic and social development in a country or region. The SDI is represented on a scale ranging from 0 to 1.^10^ The SDI 2019 value quantiles are used to categorize regions. According to their SDI 2019 values, Uttar Pradesh, Bihar, Rajasthan, Madhya Pradesh, Chhattisgarh, and Jharkhand are classified as LSDI states.

###  Study Variables

 Binary (1 if a live-born child dies before its fifth birthday; 0 otherwise) was the dependent variable. Additionally, the time in months until the event (death) occurred. Independent variables (N = 11) included the age of the mother (15–25, 26–35, and 36–49 years), educational level of the mother (no education, primary, secondary, and higher), social caste (scheduled caste [SC], scheduled tribe [ST], other backward castes [OBC], and other castes), wealth index (poorest, poorer, middle, richer, and richest), and mother’s body mass index (BMI; underweight, normal weight, overweight, and obese). Moreover, other independent variables were the gender of the child (male or female), birth order of the child (first order, second order, third order, and four or more birth orders), size of the child at birth (larger than average, average, and smaller than average), and birth weight (yes if it was less than 2.5 kg or no if it was 2.5 kg or more). Further, a separate category was created for unknown or unmeasured birth weights, and another category was used to investigate whether the child was immediately placed on the mother’s chest after birth (recorded with yes or no).

###  Statistical analysis

 Descriptive statistics (numbers and percentages) were reported for categorical variables, and the thematic map was prepared to depict the district-wise U5MR. These 248 districts were categorized into 3 groups based on U5MR. The first group encompassed districts that attained the SGD 3.2 goal, aiming at reducing under-5 mortality to at least as low as 25 per 1,000 live births (U5MR ≤ 25). The second group contained districts with U5MR between SGD 3.2 and the national average (> 25 to 41.9), and districts with U5MR more than the national average (> 41.9) included the third category. Furthermore, univariate and multivariate Cox proportional hazard (CoxPH) regressions were applied to assess the potential features. Six algorithms were applied for training, including two conventional survival models (CoxPH regression and CoxNet PH) and four models from the ML paradigm (random survival forest [RSF], gradient boosting survival [GBS], component gradient boosting survival [CGBS], and survival tree [SurvTree]). In addition, random forest (RF) permuted feature importance was employed to identify important features. The data were split into a 70:30 ratio, with 70% utilized to train the model and the remaining 30% for testing. The model was trained using the training set, and its performance was assessed on the test set to measure generalization.

 One of the widely used methods in survival analysis is the CoxPH regression.^11^ This model estimates the hazard rate (HR) for an event as a linear combination of the effects of various covariates. While this model is popular due to its simplicity and ease of interpretation, its parametric structure limits its ability to capture non-linear relationships or interactions between covariates.^12^ The CoxPH model, regularized by a convex combination of *l*_1_*(lasso)* and *l*_2_*(ridge)* penalties, is known as the CoxNet regression model.^13^

 RSF^14^ is an extension of RF designed to handle right-censored survival data. It follows the general principles of RF, where SurvTrees are grown using bootstrapped data, random feature selection is employed for splitting nodes, trees are grown deeply, and the survival forest ensemble is constructed by averaging terminal node statistics. A gradient-boosted model shares similarities with an RSF in that both utilize multiple base learners to generate overall predictions. However, they differ in their approach to combining these learners. While RSF independently fits a collection of SurvTrees and averages their predictions, gradient-boosted models build learners sequentially in a greedy, stagewise manner. The GBS^15,16^ model implements gradient boosting survival learner, whereas CGBS analysis uses component-wise least squares as a base learner. The survival tree model is a nonparametric approach designed to identify factors influencing the time until the occurrence of a specific event. The log-rank splitting rule is employed to assess the quality of a split.^17^ These models are particularly suitable for survival analysis because they can effectively handle censored data and estimate time-to-event outcomes. While the CoxPH model provides an interpretable parametric approach, tree-based models, such as RSF and gradient boosting methods, capture complex, non-linear relationships, improving predictive accuracy.

 The goodness-of-fit and model calibration of the fitted models were evaluated using the concordance index (C-index)^18,19^ and the integrated Brier score (IBS),^20^ respectively. Additionally, the time-dependent receiver operating characteristic (ROC) curve^21^ was used to assess the predictive performance of the models.

 Statistical analysis was performed using the trial version of Statistical Package for Social Sciences (SPSS) software (version 26; IBM Inc., Chicago, IL), GeoDa 1.20, R package (forester, version 0.3.0),^22^ and Python package (scikit-survival, version 0.23.1).^23^

## Results

 Among 94 202 children under the age of five in the dataset, 4284 (4.5%) were reported dead. The mothers of 44.1%, 50.2%, and 5.7% of children were in age groups of 15–25 years, 26–35 years, and 36–49 years, respectively. Moreover, 43.6% of mothers had secondary education, while 31.5% had no schooling at all. Further, 23% and 14.7% of children belonged to SCs and STs, respectively. A total of 33 952 (36%) children were from the poorest households, 23 602 (25.1%) were from poorer families, and 15 787 (16.8%) belonged to middle-income households. Overall, 12 338 (13.1%) children had mothers whose height was less than 4.9 feet. Regarding gender distribution, 52% were male, and 48% were female. Concerning birth order, 33.5%, 30.7%, and 17.6% of children were first-born, second-born, and fourth-born or later, respectively. A majority (72.6%) of children at birth were of average length, while 16.8% and 10.6% were classified as larger than average and smaller than average, respectively. A significant percentage (81.7%) of children had been placed on their mother’s chest immediately post-birth, whereas 18.3% were not (Table 1).

**Table 1:** Socioeconomic, demographic, and maternal healthcare variables determining under-five child mortality in low SDI states of India, NFHS-5 (2019-2021)

**Variables**	**Frequency **	**Percent **
Age group (years)		
15-25	41 519	44.1
26-35	47 327	50.2
36-49	5356	5.7
The mother’s highest level of education		
No education	29 675	31.5
Primary	13 338	14.2
Secondary	41 062	43.6
Higher	10 127	10.8
Caste		
Schedule caste	21 675	23
Schedule tribe	13 819	14.7
Other backword caste	45 734	48.5
Others	12 974	13.8
Wealth index combined		
Poorest	33 952	36
Poorer	23 602	25.1
Middle	15 787	16.8
Richer	12 099	12.8
Richest	8762	9.3
Mother’s height ( < 4.9 feet)		
No	81 864	86.9
Yes	12 338	13.1
Mothers’ body mass index (kg/m^2^)		
Underweight	20 788	22.1
Normal weight	61 843	65.6
Overweight	9280	9.9
Obese	2291	2.4
Gender of the child		
Male	49 027	52
Female	45 175	48
Birth order		
1	31 529	33.5
2	28 884	30.7
3	17 215	18.3
4 or more	16 574	17.6
Size of the child at birth		
Very large/larger than average	15 841	16.8
Average	68 376	72.6
Very small/smaller than average	9985	10.6
Birth weight ( < 2.5 kg)		
No	67 332	71.5
Yes	15 558	16.5
Not weighted don’t know	11 312	12
No	17 236	18.3
Yes	76 966	81.7

*Note*. SDI: Sociodemographic index; NFHS-5: National Family Health Survey-5.

**Figure 1:**
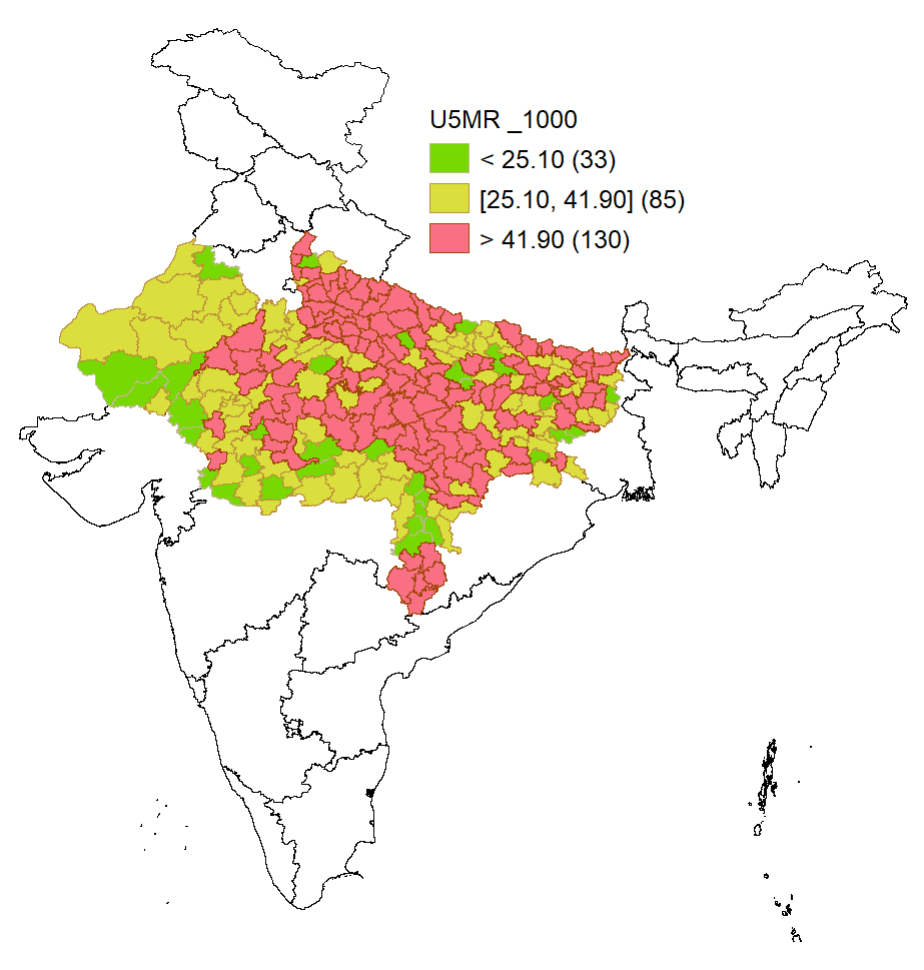
Spatial Distribution of Under-Five Mortality Rates by Districts of Low SDI States of India, NFHS-5 2019-2021. *Note*. SDI: Sociodemographic index; NFHS-5: National Family Health Survey-5

Figure 1 presents the U5MR per 1000 children across the districts of LSDI states in India. The median U5MR was 43.6 (the interquartile range: 33.25–55.32). Among the 248 districts, 32 had a U5MR below 25 per 1000, and these districts were scattered across the states. The U5MR in 85 districts ranged between 25.1 and 41.9, with the majority of these districts located in Rajasthan, Madhya Pradesh, Chhattisgarh, and parts of Bihar and Uttar Pradesh. A large number of districts (130) had a U5MR higher than the national average, with most of these districts situated in Uttar Pradesh, Bihar, Chhattisgarh, Madhya Pradesh, and parts of Rajasthan and Jharkhand.

**Figure 2:**
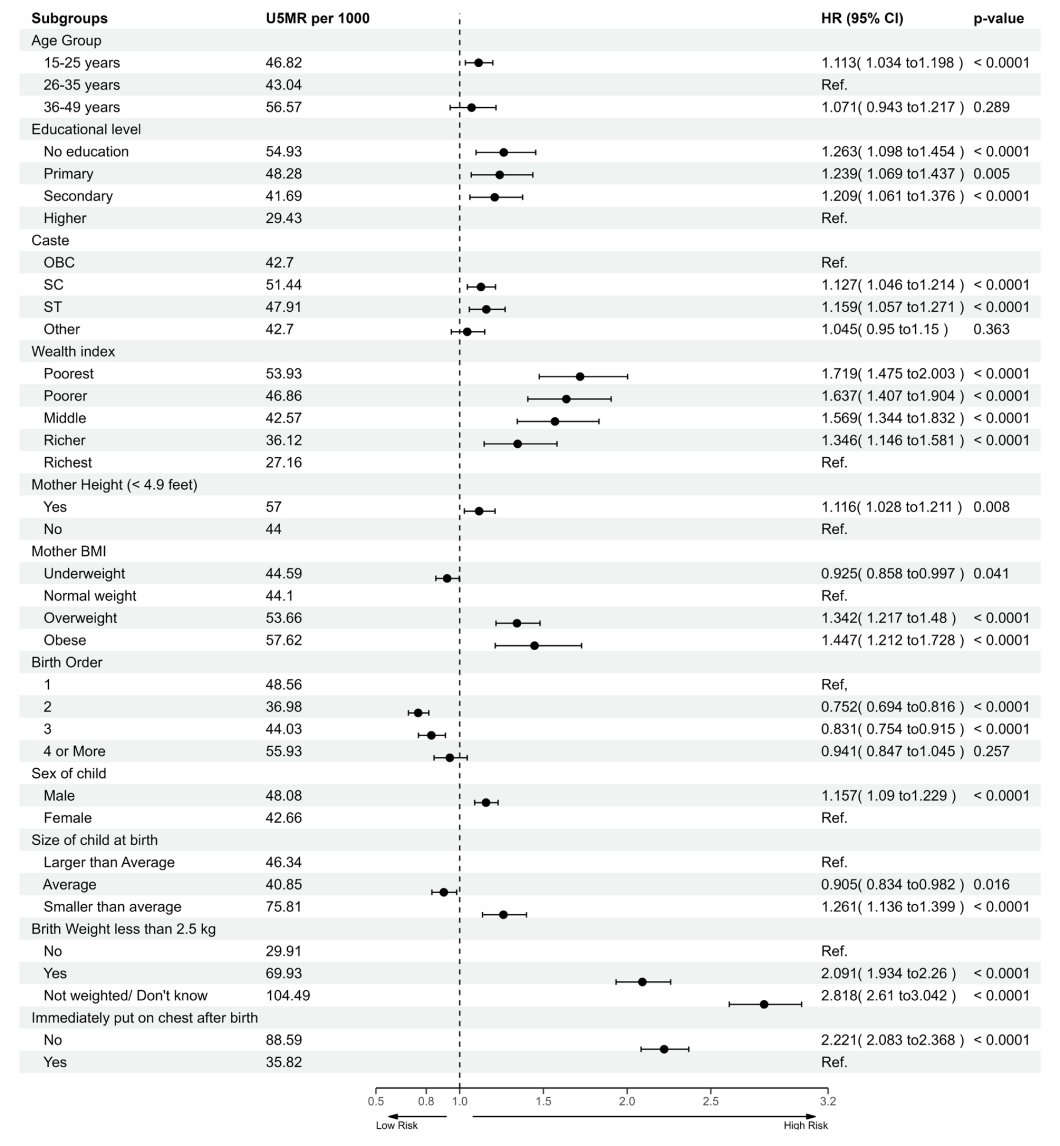
Results of the cox proportional hazard analysis highlighting factors linked to under-five mortality in India, NFHS-5 (2019–2021). *Note*. NFHS-5: National Family Health Survey-5; OBC: Other Backward Classes; SC: Scheduled caste; ST: Scheduled tribe; HR: Hazard rate; CI: Confidence interval; U5MR: Under-five mortality rate

Figure 2 shows the several significant factors associated with under-five mortality. Mothers aged 15–25 years had a higher risk of child mortality (HR = 1.113, 95% confidence interval [CI]: 1.034–1.198; *P *< 0.0001) compared to those aged 26–35 years. Lower educational status was also associated with higher child mortality risk. Mothers with no education (HR = 1.263, 95% CI: 1.098–1.454; *P *< 0.0001), primary education (HR = 1.239, 95% CI: 1.069–1.437, *P *= 0.005), and secondary education (HR = 1.209, 95% CI: 1.061–1.376; *P *< 0.0001) had elevated hazards compared to those with higher education. Caste and economic status significantly influenced child mortality. Children from SC (HR = 1.159, 95% CI: 1.057–1.271; *P *< 0.0001), ST (HR = 1.127, 95% CI: 1.046–1.214; *P *< 0.0001), and OBC (HR = 1.127, 95% CI: 1.046–1.214; *P *< 0.0001) faced higher risks.

 The risk of child mortality decreased with an increase in the wealth index. Children from the poorest households had the highest HR (HR = 1.719, 95% CI: 1.475–2.003; *P *< 0.0001), followed by poorer (HR = 1.637, 95% CI: 1.407–1.904; *P *< 0.0001) and s-income (HR = 1.569, 95% CI: 1.344–1.832; *P* < 0.0001) households. Overweight (HR = 1.342, 95% CI: 1.217–1.480; *P* < 0.0001) and obesity (HR = 1.447, 95% CI: 1.212–1.728; *P* < 0.0001) among mothers increased the risks of child mortality compared to those mothers with normal BMI. Second-born (HR = 0.752, 95% CI: 0.694–0.816; *P* < 0.0001) and third-born (HR = 0.831, 95% CI: 0.754–0.915; *P* < 0.0001) children faced lower risks of mortality compared to first-born children. Other significant findings included higher risks for male children (HR = 1.157, 95% CI: 1.090–1.229; *P* < 0.0001), children with low birth weight (HR = 2.091, 95% CI: 1.934–2.26; *P* < 0.0001), and children not immediately placed on their mother’s chest after birth (HR = 2.221, 95% CI: 2.083–2.368; *P* < 0.0001).

**Figure 3:**
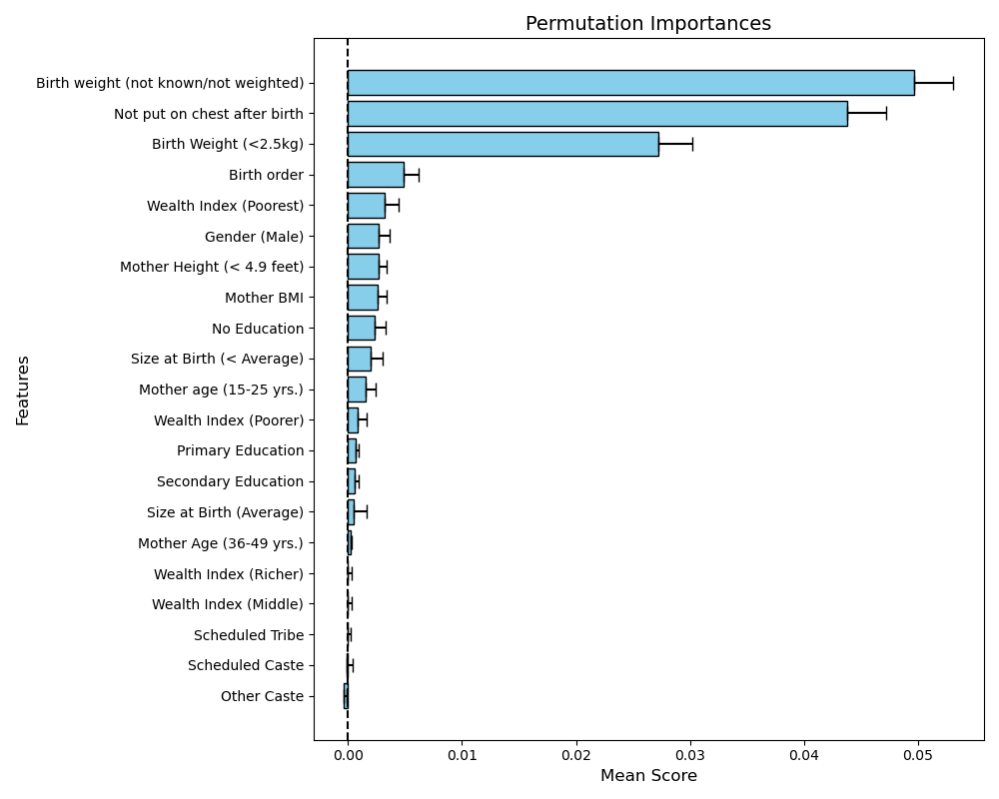
Permutation feature importance results obtained with the random forest classifier. *Note*. BMI: Body mass index

 RF permutation feature importance revealed that birth weight (not known/not weighted), lack of putting the child immediately on the chest after birth, birth weight less than 2.5 kg, birth order, and male gender were the top five features, followed by mother’s shorter height ( < 4.9 feet), mother’s BMI, lack of mother’s education, 15–25-year-old age group (of mothers), and poorer wealth index (Figure 3).

 In the ML performance matrix, the RSF model emerged as the top performer, achieving the highest C-index (0.6857) and area under the curve (AUC, 0.7125 ± 0.0189) alongside a well-calibrated IBS score of 0.0432, reflecting strong accuracy and reliability. The CoxPH and CoxNetPH models showed comparable performance with C-index scores of 0.6844 and 0.6842, IBS of 0.0430, and similar AUCs around 0.708, making them solid alternatives to RSF. The GBS model had moderate results, with a C-index of 0.6629 and AUC of 0.6996, while maintaining an acceptable IBS of 0.0435. On the lower end, SurvTree’s C-index of 0.6669 and AUC of 0.5406 indicated weaker classification abilities, though its calibration (IBS 0.0433) remained reasonable. Finally, the CGBS model was the least effective, with a C-index of 0.5787 and a low AUC of 0.5895 (Table 2).

**Table 2:** Model performance metrics based on the test dataset

**Models**	**C-Index**	**IBS**	**AUC Mean**	**AUC SD**
CoxPH	0.6844	0.0430	0.7079	0.0163
CoxNetPH	0.6842	0.0430	0.7080	0.0163
Random survival forest	0.6857	0.0432	0.7125	0.0189
Gradient boosted survival	0.6629	0.0435	0.6996	0.0193
SurvTree	0.6669	0.0433	0.5406	0.0125
Componentwise gradient boosted survival	0.5787	0.0439	0.5895	0.0105

*Note*. C-index: Concordance index; IBS: Integrated Brier score; AUC: Area under the curve; SD: Standard deviation; CoxPH: Cox proportional hazard; SurvTree: Survival tree; CoxNetPH: Cox Net proportional hazard

**Figure 4:**
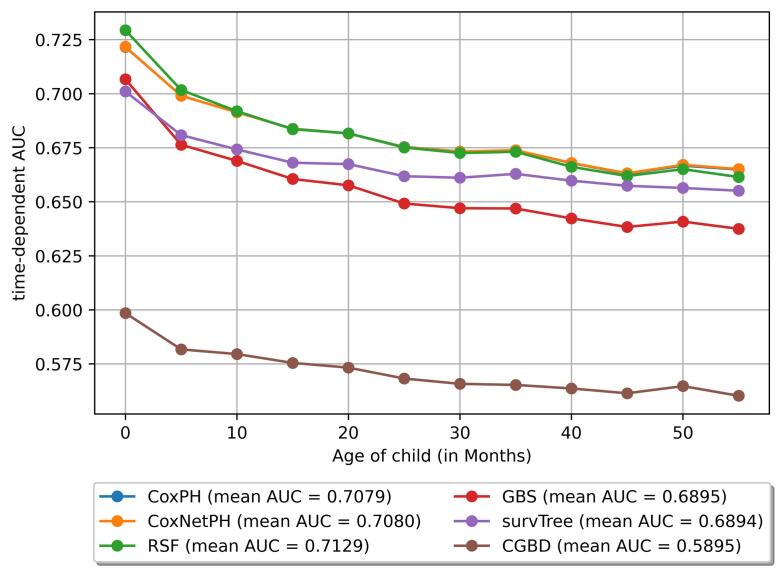
Time dependence AUC of various included models in the study. *Note*. CoxPH: Cox proportional hazard; CoxNetPH: Cox Net proportional hazard; AUC: Area under the curve; RSF: Random survival forest; GBS: Gradient boosted survival; SurvTree: Survival tree; CGBD: Component gradient boosting survival.

 Time-dependence AUC demonstrated that RSF performs slightly better on average, particularly in the first 20 months of life of children. Beyond 20 months, RSF’s advantage diminished, showing a decline in performance similar to that of the other models. The CoxPH and CoxNetPH models represented stable performance across different age groups, which is reflected in their similar average AUC scores. However, survival tree performance decreased in the first 10 months of the child’s age, and then it remained constant after 10 months. On the other hand, the GBS and CGBD models reduced, indicating a steady decline in predictive accuracy across all age ranges (Figure 4).

## Discussion

 The study investigated the under-five child mortality based on NFHS-5 data of LSDI states of India. The analysis identified key factors associated with U5MR and developed the predicting models using the traditional statistical approach and survival ML techniques. These methods provided a comprehensive understanding of the determinants of U5MR and demonstrated the potential of advanced ML techniques in predicting U5MR. The thematic map revealed significant disparities in mortality rates across districts within LSDI states of India.

 In general, 130 out of the 248 districts exhibited U5MR above the national average, highlighting the pressing need for targeted interventions in these high-burden districts to address the underlying determinants of child mortality. When compared to the SDG-3, aimed at decreasing the U5MR to 25 per 1000 live births by 2030, merely 33 districts achieved this target. These districts are scattered across the LSDI states; however, a coagulated pattern was observed in Rajasthan and Chhattisgarh. In Rajasthan, most of these districts were concentrated in the Marwar region, including Barmer, Pali, Nagaur, and Jalore, corresponding districts of Gujarat—India’s economically leading state. In contrast, in Chhattisgarh, the districts meeting the target were concentrated around the capital region, including Raipur, Durg, Bilaspur, Dhamtari, and Bemetara. Meanwhile, 85 districts represented mortality rates that, although lower than the national level, remained above the SDG-3 benchmark. These findings underscore the uneven progress made toward achieving SDG-3 within the LSDI states.

 Based on the RF permutation feature importance, low birth weight, lack of putting the child immediately on the breast after birth, birth order, poorest wealth index, male gender, maternal short stature, mother’s BMI, and lack of mother’s education were identified as important risk factors associated with child mortality. These results are in line with those of similar studies that utilized RF feature importance.^24-26^

 This study is one of the initial attempts to predict under-five child mortality using survival ML models. According to these models, the predictive analysis, the RSF demonstrates the highest C-index and time-dependent AUC, indicating strong discriminative ability. Its well-calibrated IBS of 0.0432 further supports its reliability in survival prediction. This performance underscores the robustness of RSF in handling complex survival data with non-linear and nonparametric relationships. The results conform to the findings of other studies predicting U5MR using ML classification models.^26,27^ CoxPH and CoxNetPH models had comparable C-index values (~0.684) with RSF and slightly lower AUCs (~0.708), demonstrating stable performance across different time intervals and age groups of children. These models are viable alternatives to RSF, offering consistent reliability over time. The GBS model showed moderate performance, while SurvTree achieved reasonable calibration (IBS of 0.0433) despite weaker classification ability. The CGBS model had the lowest C-index (0.5787) and AUC (0.5895), representing limited utility.

 RSF had a slight edge in the first 20 months in the time-dependent AUC-ROC curve, but its performance declined thereafter, aligning with other models. CoxPH and CoxNetPH remained stable across all age intervals, emphasizing their long-term reliability. In contrast, SurvTree revealed an initial decline but stabilization after 10 months. GBS and CGBS experienced a steady performance drop across all time frames. These results suggest that RSF is best suited for short-term to medium-term predictions, while Cox-based models excel in long-term stability, highlighting the importance of selecting models based on specific temporal and data characteristics.

 In a study using the CoxPH ratio model, it was found that several demographic, socio-economic, and maternal characteristics significantly impact the death rate of children in the first five years of life. The multivariate analysis confirmed that the risk of under-five mortality in male children was higher than in female children, which corroborates the finding of similar research conducted on NFHS-4.^28^ Maternal age is an essential factor in predicting child mortality. The health of a younger mother’s first child is negatively impacted by her biological and social factors. A child born to young mothers suffers adverse health outcomes and has an elevated risk of mortality before the age of five.^29^ A decrease in the risk of under-five mortality among children of mothers with primary education, in contrast to those of mothers without formal education, indicates that enhancing maternal education may lead to improved child survival outcomes.^30^ This expectation arises from the documented benefits of women’s educational advancements, which positively impact themselves, their children, and society as a whole.^26,31^ Educated mothers are more inclined to adopt effective health-seeking behaviour for themselves and their children, particularly in the utilization of health services, as well as in feeding and childcare practices. This, in turn, leads to improved health outcomes for both mothers and their children.^32^ The results of this study showed a negative HR between a family’s economic status and U5MR. Poor families experience higher rates of under-five mortality compared to wealthy families. This discrepancy may arise from various factors. Poor families face challenges in affording necessary maternal care and providing adequate nutrition to the mother and the child. Lack of awareness regarding overall maternal and child health care adds to these problems.^33,34^ Based on the findings of this study, the risk of mortality was higher among children from SC and ST communities in comparison to those from OBC and other castes.^33^ This could be due to inadequate postnatal care and the challenges associated with socioeconomic and cultural factors.^35^ The results of this study also demonstrated that children born with low birth weight were at a higher risk of dying before reaching the age of five compared to those with normal birth weight. A plausible reason for this could be the complications associated with preterm births. Preterm infants are more vulnerable to conditions such as sepsis, a leading cause of neonatal deaths, as well as other health issues such as neonatal jaundice and apnea. These complications collectively contribute to the increased likelihood of under-five mortality in low-birth-weight children.^36^ Children who are smaller than the average size at birth face a higher risk of mortality in comparison to those born with an average size. The findings of this study indicated that children born smaller than the average size at birth face a higher risk of mortality. Smaller-than-average newborns are more prone to malnutrition (stunting, wasting, and underweight) compared to those born with an average size.^37^ Babies not placed on their mother’s chest immediately after birth face a higher risk of mortality, with an HR of 2.207. Early skin-to-skin contact promotes breastfeeding, providing essential nutrients and antibodies that protect against infections, better cognitive development, and strengthen maternal bonding. It also helps regulate the baby’s temperature, reduces stress, and enhances maternal satisfaction, emphasizing the importance of early breastfeeding to save lives and improve well-being.^38^

 The study had several strengths. For instance, it is based on national-level data collected using validated questionnaires and methodologies. Moreover, this study, to the best of our knowledge, is one of the first attempts to predict under-five child mortality using survival ML models. However, the study had certain limitations. Considering that this study focused only on LSDI states of India, the performance of the models may exhibit variability when applied to different datasets. As a result, the findings may not be directly generalizable to other datasets. In this study, the model’s performances were measured on test data. Cross-validation was not performed. Additionally, the lack of data on the specific causes of child deaths limited our ability to evaluate unavoidable fatalities and their impact on the study’s outcomes.

**Highlights**

- This study utilized conventional and machine-learning (ML) approaches to predict child survival.
- About 4.5% of children died before reaching their fifth birthday, with higher mortality risks associated with children born to younger mothers, uneducated mothers, mothers with poor household wealth, children with low birth weight, and children with smaller than average birth size.
- The random survival forest model was most effective in identifying and assessing these risk factors, providing a robust tool for predicting child mortality.

## Conclusion

 This study compared the performance of advanced survival ML models in predicting under-five child mortality. The CoxPH model identified significant predictors in this regard, including younger maternal age, maternal education level, religion, wealth index, maternal height, child’s gender, birth order, and maternal BMI. Accordingly, the government must reinforce its commitment to reforming education and communication (IEC) initiatives currently being implemented as part of the Reproductive, Maternal, Neonatal, Child, Adolescent Health plus Nutrition package in the country, with a renewed commitment to higher budgetary allocation to Reproductive, Child, and Health Services to holistically tackle under-five mortality.

## Competing Interests

 There are no competing interests between authors regarding the research, publication, and authorship of this article.

## Ethical Approval

 The study was based on secondary data from the NFHS-5 survey, which includes no identifiable participant information. Informed consent was obtained by NFHS-5 during data collection. As the dataset is publicly accessible for research, separate ethical clearance was not necessary for this study.

## Funding

 This research received no specific grant from any funding agency, commercial entity, or not-for-profit organization.
